# Hormonal and Dietary Characteristics in Obese Human Subjects with and without Food Addiction

**DOI:** 10.3390/nu7010223

**Published:** 2014-12-31

**Authors:** Pardis Pedram, Guang Sun

**Affiliations:** Faculty of medicine, Memorial University, 300 Prince Philip Drive, St. John’s, NL A1B3V6, Canada; E-Mail: p.pedram@mun.ca

**Keywords:** food addiction, gut hormones, neuropeptides, adipokines, micro-/macro-nutrient intake

## Abstract

The concept of food addiction (FA) is a potentially important contributing factor to the development of obesity in the general population; however, little is known about the hormonal and dietary differences between obesity with and without FA. Therefore, the aim of our study was to explore potential biomarkers, including various hormones and neuropeptides, which regulate appetite and metabolism, and dietary components that could potentially differentiate obesity with and without FA. Of the 737 adults recruited from the general Newfoundland population, 58 food-addicted and non-food-addicted overweight/obese individuals (FAO, NFO) matched for age, sex, BMI and physical activity were selected. A total of 34 neuropeptides, gut hormones, pituitary polypeptide hormones and adipokines were measured in fasting serum. We found that the FAO group had lower levels of TSH, TNF-α and amylin, but higher levels of prolactin, as compared to NFO group. The total calorie intake (per kg body weight), the dietary intake of fat (per g/kg body weight, per BMI and per percentage of trunk fat) and the percent calorie intake from fat and carbohydrates (g/kg) was higher in the FAO group compared to the NFO group. The FAO subjects consumed more sugar, minerals (including sodium, potassium, calcium and selenium), fat and its components (such as saturated, monounsaturated and *trans* fat), omega 3 and 6, vitamin D and gamma-tocopherol compared to the NFO group. To our knowledge, this is the first study indicating possible differences in hormonal levels and micro-nutrient intakes between obese individuals classified with and without food addiction. The findings provide insights into the mechanisms by which FA could contribute to obesity.

## 1. Introduction

Obesity is a multifaceted condition [1] and represents a pandemic that needs urgent attention [2]. In Canada, over one in four adults are obese [3], and the province of Newfoundland has one of the highest rates of obesity in the country (after the Northwest Territories and Nunavut) [3,4]. Obesity is caused by multiple factors, including genetics, endocrine function, behavioral patterns and environmental determinants [5]. It has been well documented that chronic overconsumption of calories plays a fundamental role in the development of obesity [6]. In a previous study on the general Newfoundland population, our laboratory discovered that chronic compulsive overeating, defined as “food addiction” by the Yale Food Addiction Scale (YFAS) [7,8], significantly contributes to human obesity [9]. Additionally, the clinical symptom counts of food addiction defined by the YFAS is highly associated with the severity of obesity [9]. Addiction is considered a psychological disorder with a definite neuro-endocrine basis; however, food addiction is still not defined as an independent disorder in Diagnostic and Statistical Manual (DSM) V [10,11]. Similar to drug addiction, food addicts lose control over food consumption despite the negative consequences relevant to obesity [12,13]. This suggests that they suffer from repeated failed attempts to reduce their food intake, and they are unable to abstain from certain types of food or to reduce consumption [12].

In humans, the regulation of food intake is based on an intricate feedback system controlled by hunger and satiety signals [5,14,15]. These signals are generated in the brain, peripheral tissue and/or organs through two complementary drives, including both homeostatic and hedonic pathways [5,15,16,17]. The hedonic or reward-based regulation pathway is related to the mesolimbic dopamine pathway, which is stimulated in both drug abuse and the consumption of highly-palatable foods [15]. Evidence has shown that the release of dopamine coordinates food reward, which is impaired in food addicts [15,18]. Contrastingly, the homeostatic pathway primarily regulates the energy balance between the brain and peripheries (for instance, digestive tract and adipose tissue) [14,17,19,20]. This means that based on energy reservation and the psychological want for food, the brain increases or decreases food intake by interpreting the neuronal and hormonal signals received form peripheries [15,20,21]. Therefore, in both pathways, a large number of neurotransmitters (dopamine, cannabinoids, opioids, gamma-aminobutyric acid (GABA) and serotonin), neuropeptides (α-MSH, β-endorphin, cortisol, melatonin, neurotensin, orexin A, oxytocin and substance P, *etc.*) and hormones (gut hormones, anterior pituitary hormones and adipokines) are involved, many of which can also be detectable in serum [17,18,20,21,22,23,24,25,26,27,28,29,30]. Interestingly, many studies have linked these hormones and neuropeptides with the current obesity epidemic [21,24,31,32]. Moreover, in our previous aforementioned study on the general Newfoundland population, we have reported that food addicts consumed a higher percentage of calories from fat and protein [9]. However, to the best of our knowledge, there is no study available regarding the differences in appetite regulating hormonal level between being obese with and without food addiction.

Furthermore, macronutrients have been reported to play an imperative role in obesity, addiction-like behaviour and metabolic consequences [33,34,35]. However, there is no study available on the hormonal characteristics and potential differences of macro- and micro-nutrients between being obese with and without food addiction, which will be critical to unravel how food addiction develops. Hence, the aim of the current study is to explore potential biomarkers that may differentiate being obese with and without food addiction by measuring and comparing various hormones and neuropeptides regulating appetite and metabolism and also dietary nutrient intakes in both groups.

## 2. Experimental Section

### 2.1. Ethics Statement

This study was approved by the Health Research Ethics Authority (HREA), Memorial University of Newfoundland, St. John’s, Canada, with Project Identification Code #10.33 (latest date of approval: 21 January 2014). All participants provided written and informed consent.

### 2.2. Study Sample

The food addiction study consists of 737 subjects recruited from the general Newfoundland and Labrador (NL) population. Among them, 36 subjects met the criteria of food addiction by the Yale Food Addiction Scale. Subjects with a body mass index (BMI) of 25 kg/m^2^ or less were excluded (World Health Organization (WHO) criteria: greater than 25 is classified as overweight; over 30 is classified as obese [36]). After exclusion, 29 subjects were left for analysis. Correspondingly, 29 non-food-addicted overweight/obese (NFO) subjects were selected and matched for age, sex, BMI and physical activity. All of the subjects were part of the population CODING (Complex Diseases in the Newfoundland population: Environment and Genetics) study [37,38] and were recruited from the Canadian province of Newfoundland and Labrador using advertisements, posted flyers and word of mouth. The inclusion criteria were: (1) age >19 years; (2) born in NL with family who lived in NL for at least three generations; (3) healthy without serious metabolic, cardiovascular or endocrine diseases; and (4) not pregnant at the time of the study.

### 2.3. Anthropometric Measurements

Body weight and height were measured after a 12-h fasting period. Subjects were weighed to the nearest 0.1 (kg) in a standard hospital gown on a platform manual scale balance (Health O Meter, Bridgeview, IL, USA). A fixed stadiometer was used to measure height to the nearest 0.1 (cm). BMI was calculated by dividing participants’ weight in kilograms by the square of his/her height in meter (kg/m^2^). The subjects were classified as overweight/obese (BMI ≥ 25.00) based on BMI according to the WHO criteria [36].

### 2.4. Body Composition Assessment

Whole body composition measurements including fat mass and lean body mass were measured using dual-energy X-ray absorptiometry (DXA; Lunar Prodigy; GE Medical Systems, Madison, WI, USA). The measurements were performed in a supine position after 12 h fasting, and the total percent body fat (BF%) and percent trunk fat (TF%) were determined [37].

### 2.5. Food Addiction Assessment

The diagnosis of food addiction was based on the YFAS [7,9]. This questionnaire consists of 27 items that assess eating patterns over the past 12 months. The YFAS translates the Diagnostic and Statistical Manual IV, Text Revision (DSM-IV TR) substance dependence criteria in relation to eating behavior (including symptoms, such as tolerance and withdrawal symptoms, vulnerability in social activities, difficulties cutting down or controlling substance use, *etc.*) by applying the DSM-IV TR. The scale uses a combination of Likert scale and dichotomous scoring options. The criteria for food addiction are met when three or more symptoms are present within the past 12 months and clinically significant impairment or distress is present. The Likert scoring option is used for food addiction symptom counts (for instance, tolerance and withdrawal), ranging from 0 to 7 symptoms [7,13].

#### 2.5.1. Dietary Intakes Assessment

Macronutrients (protein, fat and carbohydrate) and 71 micronutrients intake during the past 12 months were assessed using the Willett Food Frequency Questionnaire (FFQ) [39]. Participants indicated their average use of a list of common food items, over the last 12 months. The amount of each selected food was converted to a mean daily intake value. The average daily intake for each food item consumed was entered into NutriBase Clinical Nutrition Manager (software version 9.0; CyberSoftInc, Phoenix, AZ, USA), and daily intake of macro- and micro-nutrient intakes were computed [9,40,41].

#### 2.5.2. Serum Metabolism Regulating Hormones and Neuropeptides Measurement

The concentration of a total of 34 hormones and neuropeptides were measured by magnetic bead-based quantitative immunoassay using the MAGPIX system (Millipore, Austin, TX, USA) or using enzyme-linked immunosorbent assays (ELISA) (ALISEI QS, Radim, Italy) (using morning fasting serum). Gut hormones (amylin (total), ghrelin (active), leptin, total glucagon-like peptide-1 (GLP-1), gastric inhibitory polypeptide (GIP), pancreatic polypeptide (PP), pancreatic peptide YY (PYY), connecting peptide (C-peptide) and glucagon), pituitary polypeptide hormones (prolactin, brain-derived neurotrophic factor (BDNF), adrenocorticotropic hormone (ACTH), ciliary neurotrophic factor (CNTF), follicle-stimulating hormone (FSH), luteinizing hormone (LH), growth hormone (GH) and thyroid-stimulating hormone (TSH)), adipokines (adiponectin, lipocalin 2, resistin, adipsin, plasminogen activator inhibitor-1 (PAI-1) and TNF-α) and neuropeptides (alpha-melanocyte-stimulating hormone (α-MSH), β-endorphin, cortisol, melatonin, neurotensin, orexin A, oxytocin, substance P, monocyte chemotactic protein-1 (MCP-1) and Agouti-related peptide (AgRP)) were measured in duplicate using the magnetic bead-based quantitative immunoassay with the MAGPIX system. The system was calibrated prior to each assay with the MAGPIX calibration kit, and performance was verified with the MAGPIX performance verification kit. Milliplex Analyst software was used for the analyses of data. Moreover, the concentration of fasting neuropeptide Y (NPY) was measured with the ELISA method (Millipore Corporation Pharmaceuticals, Billerica, MA, USA). All measured hormonal and neuropeptide levels were above the manufactural sensitivity. Moreover, there was no/negligible cross-reactivity between the antibodies for an analyte and any of the other analytes in these panels.

#### 2.5.3. Serum Lipids, Glucose and Insulin Measurement

Concentrations of serum total cholesterol, high-density lipoprotein (HDL) cholesterol, triacylglycerols (TG) and glucose were analyzed using Synchron reagents with an Lx20 analyzer (Beckman Coulter Inc., Fremont , CA, USA). Low-density lipoprotein (LDL) cholesterol was calculated by the following: total cholesterol-HDL-TG/2.2. Serum insulin was evaluated using an immunoassay analyzer (Immulite; DPC, Los Angeles, CA, USA). Additionally, the serum insulin level was measured using an immunoassay analyzer (Immulite; DPC, Los Angeles, CA, USA) [42,43].

#### 2.5.4. Physical Activity Assessment and Other Covariates

The Baecke physical activity questionnaire was used to assess physical activity. This questionnaire assesses physical activity using three indices, including work, sport and leisure. All participants completed forms to screen medical history, demographics (gender, age and family origin), disease status, cigarette usage and medication use [44,45].

### 2.6. Statistical Analysis

All statistical analyses were completed using SPSS, version 19.0 (SPSS Inc., Chicago, IL, USA). Data are presented as the mean ± standard deviations (SD). Student’s *t*-test analyses were employed to investigate the differences in measured variables between food addicted and non-food-addicted obesity. For all analyses, statistical tests were two-sided and the alpha level was set at 0.05.

## 3. Results

### 3.1. Physical Characteristics and Fasting Serum Lipids, Glucose and Insulin Level

Demographic, fasting serum lipids, glucose and insulin level and physical characteristics of the participants are presented in Table 1 (adiposity is based on BMI). There were no significant differences for the aforementioned variables between the food-addicted overweight/obese (FAO) and NFO groups.

### 3.2. The Comparison of Metabolism Regulating Hormones and Neuropeptides in FAO and NFO

Serum hormonal levels were compared between the food addiction overweight/obese and non-food addiction overweight/obese groups (Table 2). The FAO group had a significantly lower level of amylin, TNF-α and TSH and a higher level of prolactin, as compared to the NFO group (*p* < 0.05).

**Table 1:** Characteristics of the study participants *.

Variables		NFO (Mean ± SD)	FAO (Mean ± SD)
Number		29	29
Age (year)		42 ± 8.9	42.5 ± 9.4
Sex	F	24	24
	M	5	5
BMI (kg/m^2^)		32 ± 4.42	32.5 ± 6
BF%		42.32 ± 6.4	42.7 ± 7.8
TF%		45.1 ± 5.3	46.2 ± 7.1
Physical activity		7.1 ± 1.3	7.3 ± 1.1
Glucose (mmol/L)		5.2 ± 1	5.3 ± 0.8
Cholesterol (mmol/L)		5.3 ± 1	4.9 ± 1.3
TG (mmol/L)		1.4 ± 0.9	1.3 ± 0.7
HDL (mmol/L)		1.3 ± 0.3	1.4 ± 0.3
LDL (mmol/L)		2.9 ± 1.1	3.3 ± 1.0
Albumin (g/L)		39.5 ± 3.3	39.1 ± 2.9
Insulin (pmol/L)		90.4 ± 101.9	95.9 ± 139.9

* Mean ± standard deviation (SD); BMI, body mass index; BF%, percent body fat; TF%, percent trunk fat; TG, triglycerides; HDL, high-density lipoprotein cholesterol; LDL, low-density lipoprotein cholesterol; FAO, food-addicted overweight/obese; NFO, non-food-addicted overweight/obese was defined by BMI according to World Health Organization (WHO) criteria [36].

**Table 2:** Hormonal and neuropeptide characteristics in FAO and NFO *.

Hormones		FAO	NFO	*p* **
		Mean ± SD (14–29)	Mean ± SD (14–29)	
Neuropeptides	NPY (pg/mL)	8.81 ± 3.74	5.71 ± 3.82	0.65
	α-MSH (pg/mL)	148.06 ± 84.16	147.2 ± 89.12	0.88
	β-Endorphin (pg/mL)	377.86 ± 90.82	396.54 ± 108.3	0.48
	Cortisol (pg/mL)	230,056 ± 100,323	232807.9 ± 138,900	0.09
	Melatonin (pg/mL)	3320.9 ± 1377.7	3652.75 ± 1652.43	0.65
	MCP1 (pg/mL)	294.43 ± 88.2	282.56 ± 90.11	0.83
	Neurotensin (pg/mL)	379.6 ± 103.05	379.32 ± 100.7	0.84
	Oxytocin (pg/mL)	119.5 ± 49.13	120.22 ± 57.86	0.78
	Orexin A (pg/mL)	969.6 ± 438.2	974.5 ± 347.5	0.28
	AGRP (pg/mL)	16.11 ± 6.94	16.18 ± 8.26	0.88
	Substance P (pg/mL)	39.16 ± 12.51	39.7 ± 15.06	0.53
Gut hormones	Amylin (pg/mL)	24.9 ± 11.3	32.05 ± 18.75	0.04
	GLP-1 (pg/mL)	19.91 ± 22.54	21.4 ± 22.1	0.10
	Ghrelin (pg/mL)	25.4 ± 15.8	25.91 ± 17	0.9
	Leptin (pg/mL)	20795.4 ± 12173.3	18206.72 ± 10765.9	0.50
	GIP (pg/mL)	17 ± 16.31	17.05 ± 12	0.90
	Glucagon (pg/mL)	22.61 ± 10.5	45.1 ± 52.02	0.77
	PP (pg/mL)	49.3 ± 79.4	46.85 ± 53.4	0.50
	PYY (pg/mL)	68.33 ± 122.3	93 ± 109.3	0.45
	C-peptide (pg/mL)	1373.7 ± 740.15	1269 ± 506.74	0.50
Pituitary polypeptide hormones	Prolactin (pg/mL)	2335.1 ± 1197.8	1938.3 ± 745.5	0.02
	ACTH (pg/mL)	3.05 ± 2.56	5.55 ± 6.93	0.12
	BDNF (pg/mL)	2219.3 ± 658.73	2138.38 ± 931.52	0.17
	LH (mlU/mL)	6.2 ± 7.3	6.21 ± 8.6	0.93
	FSH (mlU/mL)	13.27 ± 18.75	9.58 ± 14.12	0.35
	GH (pg/mL)	505.76 ± 635.84	810.83 ± 1019.56	0.07
	TSH (μlU/mL)	0.23 ± 0.32	1.1 ± 2.14	0.01
	CNTF (pg/mL)	148.1 ± 324.22	1647.4 ± 6280.6	0.06
Adipokines	TNF-α (pg/mL)	4.21 ± 1.23	4.5 ± 2.2	0.02
	Adiponectin (pg/mL)	65700.8 ± 68327.1	71437.3 ± 56215.3	0.71
	Lipocalin (pg/mL)	357 ± 151.7	462.2 ± 153	0.71
	Adipsin (pg/mL)	7167.66 ± 2888.25	8009.9 ± 2733	0.86
	PAL1 (pg/mL)	261.3 ± 88.8	261.31 ± 88.84	0.80
	Resistin (pg/mL)	82 ± 43.4	109 ± 55.8	0.33

* Mean ± standard deviation (SD); FAO, food-addicted overweight/obese; NFO, non-food-addicted overweight/obese; NPY, neuropeptide Y; α-MSH, alpha-melanocyte-stimulating hormone; MCP1, monocyte chemotactic protein-1; AGRP, agouti-related peptide; GLP-1, glucagon-like peptide-1; GIP, gastric inhibitory polypeptide; PP, pancreatic polypeptide; PYY, pancreatic peptide YY; ACTH, adrenocorticotropic hormone; BDNF, brain-derived neurotrophic factor; LH, luteinizing hormone; FSH, follicle-stimulating hormone; GH, growth hormone; TSH, thyroid-stimulating hormone; CNTF, ciliary neurotrophic factor; PAL1, plasminogen activator inhibitor-1; ** The independent *t*-test was set to *p* < 0.05. Some significant results would be no longer significant if multiple corrections were performed.

### 3.3. Comparison of Macronutrients and Micronutrients Intake between FAO and NFO Groups

Total calorie intake and macronutrients consumed expressed in absolute grams and in gram per kg of body weight, BMI, %BF and %TF are shown in Table 3. Total calorie intake per kg of body weight was significantly higher in the FAO group. The amount of carbohydrate intake per kg of body weight, fat consumed (per kg body weight, per BMI, per percentage of trunk fat) and the percent calorie intake from fat were significantly higher in food-addicted obesity as compared to non-food-addicted obese subjects (*p* < 0.05).

In addition, micronutrient intakes expressed as gram per kg body weight were compared between the two groups (Table 4). In general, FAO consumed significantly higher amounts of dietary sugar, mineral substances, including sodium, potassium, calcium and selenium, fat, saturated fat, trans fat, monounsaturated fat, omega 3, omega 6, vitamin D and gamma-tocopherol than the NFO group.

**Table 3:** Macronutrient intake characteristics in food addiction and non-food addiction overweight/obese groups *.

Macronutrients		FAO (*n* = 29)	NFO (*n* = 29)	*p*
		Mean ± SD	Mean ± SD	
Calorie intake	Per person	2077.4 ± 687.6	1714.0 ± 612	0.7
	per kg body weight	24.4 ± 10.9	19.5 ± 6.6	0.02
	per BMI	66.2 ± 26.5	54.1 ± 19.5	0.3
	per BF%	50 ± 16.4	42.0 ± 19.4	0.7
	per TF%	45.6 ± 14.8	38.6 ± 15.5	0.8
Fat (g)	Per person	63.6 ± 26.3	45 ± 15.6	0.054
	per kg body weight	0.7 ± 0.4	0.5 ± 0.2	0.004
	per BMI	2 ± 0.9	1.4 ± 0.5	0.01
	per BF%	1.5 ± 0.7	1.1 ± 0.5	0.1
	per TF%	1.4 ± 0.6	1 ± 0.4	0.04
	percent calorie	27.1 ± 7.5	23.4 ± 4	0.005
Carbohydrate (g)	Per person	273 ± 103	246.3 ± 93	0.6
	per kg body weight	3.2 ± 1.6	2.8 ± 1	0.03
	per BMI	8.7 ± 3.9	7.8 ± 3	0.2
	per BF%	6.5 ± 2.4	6.01 ± 2.8	0.6
	per TF%	6 ± 2.2	5.5 ± 2.3	1
	percent calorie	51.2 ± 7.1	56.3 ± 5.2	0.3
Protein (g)	Per person	99 ± 29	79.2 ± 30.8	0.8
	per kg body weight	1.1 ± 0.4	0.9 ± 0.3	0.2
	per BMI	3.1 ± 1.1	2.5 ± 1	0.3
	per BF%	2.4 ± 0.7	1.9 ± 0.9	0.8
	per TF%	2.2 ± 0.6	1.8 ± 0.7	0.9
	percent calorie	19.3 ± 3.9	18.2 ± 2.6	0.2

* Mean ± standard deviation (SD); FAO, food-addicted overweight/obese; NFO, non-food-addicted overweight/obese; BMI, body mass index; BF%, percent body fat; TF%, percent trunk fat. The independent *t*-test was set to *p* < 0.05.

**Table 4:** Significant differences of selected micronutrient intakes between food addicts (FAO) and non-food addicts (NFA) of overweight/obese groups *.

Micronutrient Intake	FAO (*n* = 29)	NFO (*n* = 29)	*p*
	Mean ± SD	Mean ± SD	
Sugar (g/kg)	1.4 ± 0.8	0.2 ± 0.5	0.03
Saturated fat (g/kg)	0.3 ± 0.1	0.2 ± 0.1	0.01
Trans fat (mg/kg)	1.0 ± 0.0	0.1 ± 0.0	0.01
Monounsaturated fat (g/kg)	0.3 ± 0.1	0.2 ± 0.1	0.01
Poly-saturated fat (g/kg)	0.1 ± 0.1	0.1 ± 0.0	0.0
Omega 3 (mg/kg)	7.0 ± 0.0	5.0 ± 0.0	0.01
Omega 6 (g/kg)	0.1 ± 0.0	0.03 ± 0.0	0.0
Vitamin B1 (mg/kg)	0.02 ± 0.01	0.02 ± 0.0	0.04
Vitamin D (IU/kg)	2.5 ± 2.1	1.9 ± 1.0	0.04
Dihydrophylloquinone (mcg/kg)	0.3 ± 0.0	0.2 ± 0.0	0.03
Gamma tocopherol (mg/kg)	0.3 ± 0.0	0.0 ± 0.0	0.04
Sodium (mg/kg)	26.1 ± 12.0	19.4 ± 6.3	0.01
Calcium (mg/kg)	13.0 ± 7.1	10.0 ± 4.0	0.02
Potassium (mg/kg)	50.8 ± 21.3	41.2 ± 16.8	0.04
Selenium (mg/kg)	1.4 ± 0.6	1.1 ± 0.3	0.02

* Mean ± standard deviation (SD); FAO, food-addicted overweight/obese; NFO, non-food-addicted overweight/obese. The independent *t*-test was set to *p* < 0.05.

## 4. Discussion

In general, endocrine factors have an important role as appetite regulating signals. A large number of hormones play a role in feeding regulation [15,16,17,24]. The abnormality in the aforementioned hormonal secretions can lead to overeating and, consequently, obesity [16,24]. Interestingly, similarities in hormonal changes have been found between obesity and substance abuse addiction [10,18]. According to the etiology, obesity is a complex disease and can be caused by many genetic and environmental factors. As we previously reported, food addiction may be an important factor leading to obesity with a unique etiology [9]. To the best of our knowledge, this study is the first to attempt to prove the idea that obesity with a definite food addiction may manifest distinguished dietary intake and hormonal characteristics.

The first finding in the current study was the significantly lower serum level of TSH and the higher level of prolactin in obese food addicts as compared to obese non-food addicts. Several population-based studies have shown a significant association of BMI with TSH and prolactin levels [46,47,48,49,50]. Findings from our current study indicate that the combined abnormality of TSH and prolactin might be one of the hormonal characteristics in obesity with food addiction rather than in general obesity. Data from a number of studies have suggested that the serum TSH level may be a marker of alcohol, opium and cocaine dependence and craving [51,52,53]. A significant negative correlation between TSH level and alcohol craving has been reported in alcohol-dependent subjects [51], and a significantly lower level of TSH has been found in opium users as compared to healthy controls [54]. Taken together with our present findings, a lower level of circulating TSH is not only associated with alcohol, opium and cocaine dependence, but also with food addiction. The significant association of prolactin in obese food addicts and the data from other studies on alcoholics, heroin and cocaine addicts with elevated basal prolactin [51,55,56,57,58] strongly suggests the involvement of circulating prolactin with food addiction, as well.

Another significant finding in the current study is the significant lower level of serum TNF-α in the obese food addiction group as compared to the obese non-food addiction group. TNF-α level is usually higher in the obese people compared to healthy controls [59]. TNF-α is known as an anorexigenic cytokine, which reduces food intake. It is thought that the impaired actions of TNF-α may lead to obesity [32]. It was reported that the levels of circulating TNF-α have been altered in alcoholics, cocaine abusers and opiate addicts. In addition, it has been suggested that TNF-α can be a potential diagnostic biomarker for drugs of abuse [60,61,62,63,64,65]. In an animal model, TNF-α has been investigated as a potential therapeutic target to prevent drug abuse and to increase the chance of cessation. [61]. The current findings of the association of low TNF-α with food addiction is very interesting and unique. There is more likely a specific manifestation in obese food addicts contrary to the increased level of TNF-α in obese people.

In the current study, we also measured serum neuropeptides regulating appetite. Neuropeptides are predominately synthesized and secreted in the central nervous system; however, levels of some neuropeptides can be detected in the peripheral circulation system [22,23,25,26,27,28,29,30]. Abnormalities of neuropeptide levels have also been found in individuals with other addictions and obesity [66,67,68,69,70]; however, in this study, no significant differences in the level of any of the measured neuropeptides were found between food addicted and non-food addicted obese subjects.

The third important finding in the current study was the significantly lower level of serum amylin in obese food addicts compared to the obese non-food addicts. This seems to be the first report regarding the link of amylin with food addiction or any other types of addictions. It is not clear at this stage if this low level of circulating amylin is a reflection of food addiction status or simply is just a secondary change owing to other factors. In a randomized crossover study on 10 healthy males consuming one meal high in carbohydrate or fat, it has been shown that amylin is affected by the macronutrient compositions of a meal, as the amylin level was greater after a high carbohydrate meal compared to a high fat meal [71]. In this study, dietary fat intake was higher in obese food addicts, which may be at least partially responsible for the low level of serum amylin.

In our previous study, we found that all food addicts, regardless of obesity status, consumed a higher percentage of calories from fat [9]; the same result was also found in an obese food addicts cohort. The high intake of dietary fat was further supported by the finding showing that obese food addicts consumed higher total calories per kilogram of body weight, higher carbohydrates per kilogram of body weight and dietary fat per kilogram of body weight (and per BMI and per percentage of trunk fat). For the first time, we also explored the potential differences of 71 micronutrients intake between food-addicted and non-food-addicted obese subjects. Corresponding to our previous discovery, we found that obese food addicts consumed a significantly higher amount of fat subcomponents: saturated, monosaturated, poly-saturated and trans fat, omega 3 and 6, vitamin D, gamma tocopherol and dihydrophylloquinone (the main source in commercially-baked snacks and fried food [72]) compared with obese non-food addicts. In addition, obese food addicts consumed higher amounts of sodium and sugar. Therefore, taken together, the data suggest that obese food addicts may consume more hyper-palatable foods that are known to have high amounts of fat, sugar and salt (sodium).

In the present study, the YFAS and Willett Food Frequency Questionnaire (FFQ) were used as tools for the diagnosis of food addiction and measuring nutrient intake over the past 12 months. These sets of measures and the criteria on which they are based have been validated in different populations [7,39,40,41,42,43,44,45,46,47,48,49,50,51,52,53,54,55,56,57,58,59,60,61,62,63,64,65,66,67,68,69,70,71,72,73,74,75,76]. The YFAS is the only tool available for the diagnosis of food addiction. Using this set of criteria can help to distinguish subjects who regularly indulge in hyper-palatable foods from those who have lost control over their eating behaviour [7,9]. However, since the aforementioned questionnaires are self-reported, there tends to be self-reporting bias.

It needs to be indicated that food addiction is a complex disease, and numerous factors are involved in the etiology. Psychological conditions, like anxiety and depression, which may cause the fluctuation of TSH, prolactin and TNF-α, were not assessed in the current study [77,78,79,80,81,82,83,84]. A related study showed that in alcohol-dependent patients, it has been shown that the hypothalamic-pituitary thyroid axis may have the ability to lead to anxious or depressed mood, which may further affect the TSH level [51].

In the current study, the active form of ghrelin was measured. However, the specific inhibitor was not added during sample collection, and therefore, it cannot be excluded that part of the ghrelin may have been degraded. Since all of the samples after blood drawing were placed immediately on ice during the entire process of all experiment, we believe that any degradation would be little, because enzymes that degrade ghrelin would have little activity at this ice-cold temperature.

The correction for multiple comparisons has not been made, since this study is a pioneering study and numerous markers were measured. Moreover, the sample size is relatively small in both groups. However, each of the individuals were well matched in both groups for gender, age, BMI and physical activity level, which would reduce the heterogeneity of subjects and increase the statistical power to detect possible difference in most variables between the two groups. Nonetheless, larger cohorts in different populations are warranted to replicate our findings.

## 5. Conclusions

To the best of our knowledge, this is the first study that has discovered significant differences in multiple aspects, including hormonal levels and nutritional intakes, between obese food addicts and obese non-food addicts. The findings provide valuable evidence to promote further understanding of the mechanism of food addiction and its role in the development of human obesity.
